# Two gas metal arc welding process dataset of arc parameters and input parameters

**DOI:** 10.1016/j.dib.2021.106790

**Published:** 2021-01-29

**Authors:** Rogfel Thompson Martinez, Guillermo Alvarez Bestard, Sadek C. Absi Alfaro

**Affiliations:** a Postgraduate Program in Mechatronic System (PPMEC), Student grant CAPES, University of Brasilia, Brazil; b Electronics Engineering, Campus Gama, University of Brasilia, Brazil; c Department of Mechanical Engineering, University of Brasilia, Brazil

**Keywords:** Deep learning, Dynamic drop volume, GMAW Process, Machine learning

## Abstract

The dataset was collected from experiments using the gas metal arc welding (GMAW) process. The experiments were planned with Central Composite Design to obtain a greater variety of data. This variability helps to develop a predictive model more generalistic with machine learning techniques. It was collected welding arc images and weld bead geometry images. Welding arc images were processed with a deep learning technique to detect drop detachment and short circuit transfer mode. These detections were useful to calc drop detachment frequency, short circuit frequency, and molten volume in every moment of GMAW process time. It was obtained the weld bead geometry parameters by process time too. All these data, joining input parameters were correlated, resulting in the datasets shown in this article.

## Specifications Table

**Table:** 

Subject	Mechanical Engineering and Applied Machine Learning
Specific subject area	Data analysis, Images Processing and Welding Process
Type of data	Table Image
How data were acquired	The images were captured with a high-speed camera and the technique uses to visualize the welding arc was Shadowgraphy.
	The welding arc parameters were obtained with a deep learning model developed in PyTorch and python script to processing and calculated them
Data format	Raw
	Analyzed
Parameters for data collection	The Design of Experiments involving two different experiments of GMAW processes. Its parameters were exposed in subsection: Experiments design.
Description of data collection	The data used for the analysis was gotten from two experimental processes. It was obtained welding arc images of 512 ×/512 pixel and weld bead geometry images to collect geometry parameters. The other description of data collected were exposed in subsection: Data Description.
Data source location	Institution: University of Brasilia
	City/Town/Region: Brasilia
	Country: Brazil
Data accessibility	Mendeley Data https://doi.org/10.17632/2nyjpb89bf.1

## Value of the Data

• This data collection can be useful to create and analyze GMAW process models with machine learning techniques. This data collection can be useful to join it to other data collection for more extensive research about GMAW process.
• This data collection can benefit any GMAW process researcher that wants to apply artificial intelligence techniques to monitoring, analyze, or control this process.
• These datasets can be used/reused for explainability and interpretability analysis, for modeling of GMAW process.

## 1 Data Description

GMAW (Gas Metal Arc Welding) is a consumable electrode welding process that produces an arc between weld pool and a continuously supplied filler metal. This welding process is considered a highly non-linear, multivariable, coupled and time-varying system [1], [2]. GMAW is widely used industrially and can be applied in ferrous and non-ferrous materials. This is mainly due to its versatility, relatively high productivity, reliability and ease of use and automation [3], [4], [5]. GMAW parameters are called all the variables involved in the welding process, whose changes will have influences on the characteristics of heat and metal transfer and finally on weld-bead geometry [6]. GMAW process offers some difficulty correctness of the welding parameters, mainly due to the relatively high number of variables and, above all, a strong interrelation between these [5], [7]. In this paper, some parameters are selected for GMAW process datasets.

### 1.1 Raw data

Some raw data, present in this article, was captured from the welding arc:

• Electric arc current: it is the electric current between electrode and weld pool in GMAW process. The file Corr_00[1,2].lvm contains these experiments values.
• Arc voltage: it is the voltage between electrode and weld pool in GMAW process. The file tensao_00[1,2].lvm contains these experiments values.
• Arc images: they are images taken of the welding arc with shadowgraphy technique. The file img_exp[1,2].tar.gz contains these images.

### 1.2 Weld bead geometry

**Fig. 1:**
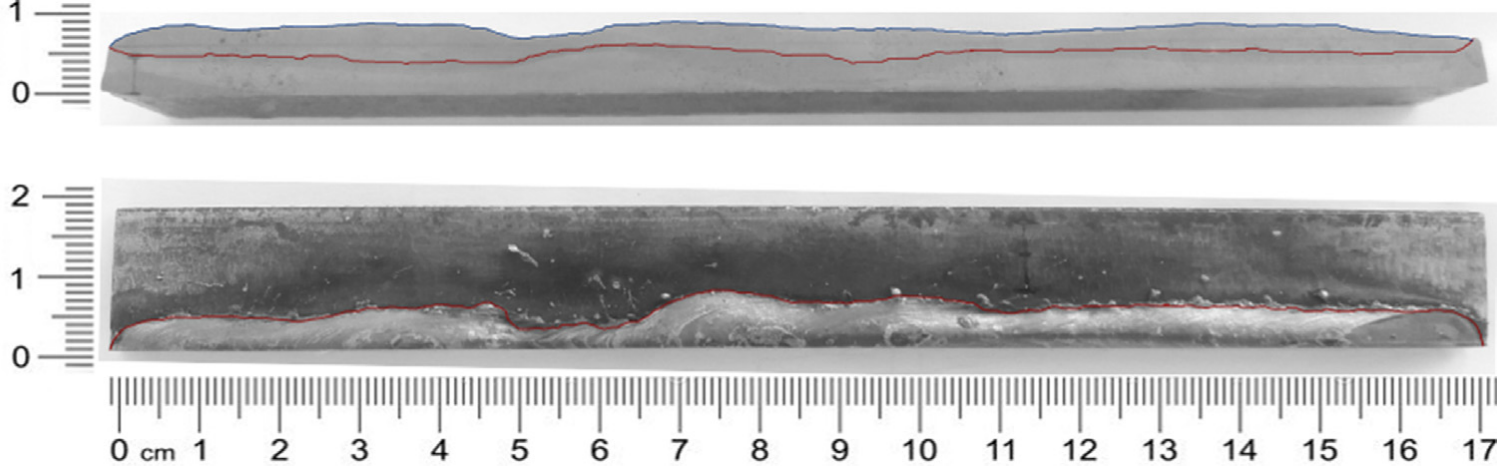
Algorithm of Weld bead geometry calculation.

Photos were taken of the resulting weld bead geometry to obtain the geometric parameters and complete the dataset. Fig. 1 shows one example. The get_geometry.py file contains the algorithm to generate the dataset of geometric parameters.

### 1.3 Volume dataset characteristics

It was generated a dataset of 2170 records, taking of 2 experiments. The Table 1 shows a summary of the dataset formed. The rows $wire_{v},$ $weld_{v},$ and voltage represent wire-rate speed, welding velocity, and voltage respectively. They are input parameters of GMAW process, controlled by the welding source. The rows volume and $wire_{l}$ are the molten volume and unmelted wire length calculate with the techniques describes in Molten or drop volume calculation section. They are arc parameters of GMAW process. The rows depth, height, and width are the weld bead geometry parameters collected. They are output parameters of GMAW process. The column main represents average values, columns min and max are minimum and maximum values of each variable. The column std is the standard deviation over the requested variable.

**Table 1:** Volume dataset description.

	main	std	min	max
$wire_{v}\left(m/min\right)$	6.38	1.06	4.80	8.20
voltage(volt)	26.09	3.63	17.00	32.00
$weld_{v}\left(mm/s\right)$	9.99	1.90	6.20	13.40
$wire_{l}\left(mm\right)$	13.78	1.12	9.00	15.00
$volume(mm^{3})$	0.74	0.98	$5.6\times 10^{-5}$	10.54
depth(mm)	1.95	0.89	0.00	3.62
height(mm)	1.77	0.86	0.00	3.33
width(mm)	5.94	1.28	2.67	8.21

### 1.4 Frequency dataset characteristics

The frequency dataset was created for can do a comparative analysis of the resultant models both frequency dataset and volume dataset. They differ in the arc parameters analyzed. The frequency dataset removes some parameters, like volume and unmelted wire length, and add dropfreq. Table 2 shows a summary of dataset formed. The row dropfreq represent short circuit and drop frequency detected on 50ms. This dataset has 585 records.

**Table 2:** Frequency dataset description.

	main	std	min	max
dropfreq(unit)	3.68	3.82	1.00	24.00
$wire_{v}\left(m/min\right)$	6.42	1.05	4.80	8.20
voltage(volt)	25.17	4.09	17.00	32.00
$weld_{v}\left(mm/s\right)$	10.06	1.90	6.20	13.40
depth(mm)	1.97	0.86	0.00	3.62
height(mm)	1.97	0.74	0.00	3.33
width(mm)	5.91	1.29	2.67	8.21

## 2 Experimental Design, Materials and Methods

It was obtained arc GMAW process images in experiments carried out with the following equipment:

• Welding power source: fully digitized microprocessor-controlled inverter power sources [8]. The manipulation of the power source is done with a computer through an interface.
• Software of welding power source and table: This software is responsible for the communication with interface, which at the same time communicates the computer with the welding source. It controls the welding table too.
• Welding table: The linear table, developed in GRACO lab, Brasilia University, is a platform in which the piece to be welded is placed and secured and that can move linearly in one direction. The displacement of the part is transmitted by a stepper motor through an auger. The speed, direction, and travel time of the table are adjusted, allowing control of the start and end of the weld beads [9].
• High-speed camera: it provides megapixel resolution images at frame rates up to 3000 frames per second (fps), 512 ×/512 pixels(px) resolution at 10,000 fps. In these experiments are used 1000 fps and 1024 px resolution.

The materials used in this study were:

• Wire electrode: wire with 1,2 mm of diameter.
• Base material: 1020 steel in flat sheet format, dimensions 6,35 mm thick, and 300 mm ×/40 mm long and width respectively.
• Shielding gas: it is used 96% argon and 4% carbon dioxide.

### 2.1 Experiments design

The process of each experiment lasted 20 seconds. Experiments data were planned with the factorial design of Central Composite Design [10], because it generates a reasonable data distribution, with long possible distribution in the dataset. The objective is to obtain the greatest possible variety of data for better computational model creation. This result was demonstrated with the datasets obtained. These Tables 3 and 4 show the experimental data distribution.

**Table 3:** Experiment 1: data distribution.

time	wire rate	voltage	welding speed
(s)	(m/min)	(volt)	(mm/s)
0	5.5	20	8
3	5.5	20	8
5	7.5	20	8
7	5.5	29	8
9	7.5	29	8
11	5.5	20	12
13	7.5	20	12
15	5.5	29	12
17	7.5	29	12

**Table 4:** Experiment 2: data distribution.

time	wire rate	voltage	welding speed
(s)	(m/min)	(volt)	(mm/s)
0	4.8	24.5	10
3	4.8	24.5	10
5	8.2	24.5	10
7	6.5	17	10
9	6.5	32	10
11	6.5	24.5	6.6
13	6.5	24.5	13.4
15	6.5	24.5	10
17	6.5	24.5	10

### 2.2 Weld bead geometry analysis

On the other hand output data that complete the dataset are weld bead geometry data (width, depth, and height) of experimental result. The weld bead geometry was obtained from the macrographic analysis. The macrographic analysis was made in a longitudinal direction, this is in the direction of the torch movement. In these cases, it is taken the maximum value in each measurement point like express [11]. To obtain weld bead geometry data was necessary polished and etched using 2.5% nital solution to display the weld bead penetration. With all this information, in the next phase, it proceeds to obtain information that shows these images. All data were analyzed after 3 seconds when the process was more stable. The dimensions of the weld bead geometry were obtained, using an image processing algorithm. The algorithm was applied to convert the values in respective millimeters dimensions. For calculations of this algorithm, it is necessary to know the dimensions of the piece on right and left in pixels and millimeters.

### 2.3 Molten or drop volume calculation

In the GMAW process area, several equations have been investigated for the calculation of the molten volume, but [12] is a simple and agile way to be applied in this algorithm. It necessary the drop detachment or short circuit detection in a GMAW process by image sequences and detection time. Background subtraction techniques can help in this. Background subtraction is a widely used approach for detecting moving objects in videos from static cameras between two consecutive frames [13]. This is a simple method to detect movement [13], [14]. The frame difference used in this work is:

(1) $$F\left(t\right)=I_{t-2}-I_{t}/3$$

**Fig. 2:**
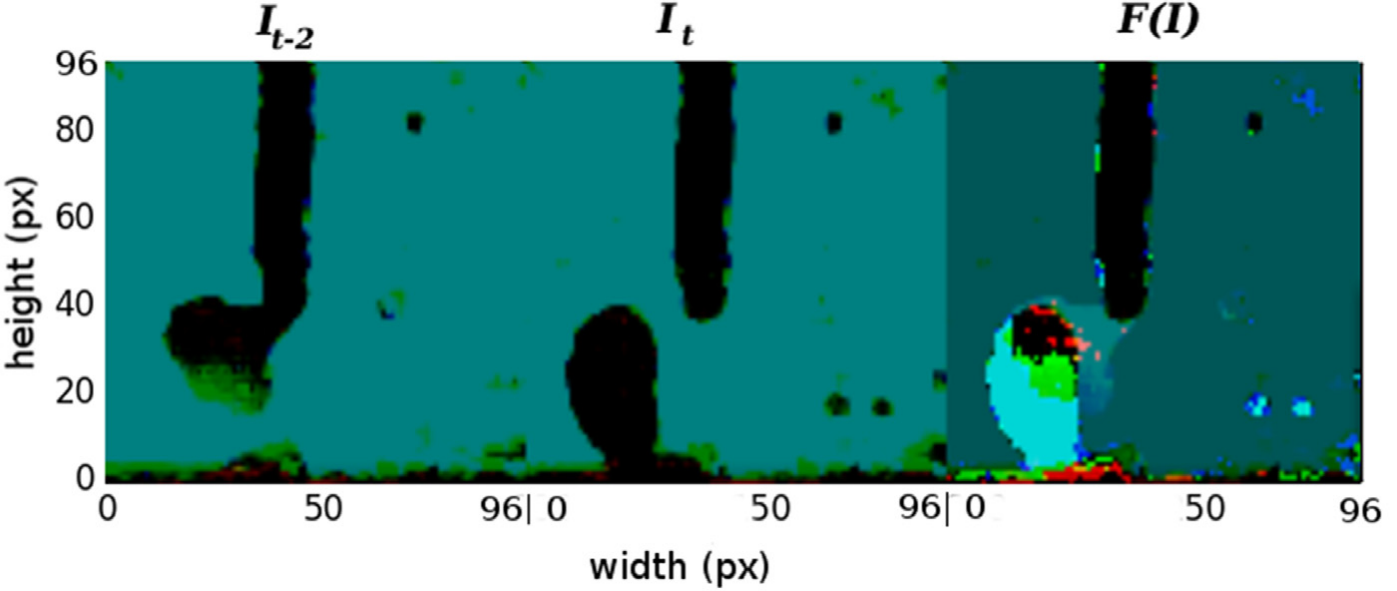
Images example: drop detachment pattern [15].

Where $I_{t}$ is the image on time equal t, the division by 3 aims to keep visible image element. The final pattern is shown Fig. 2.

This pattern was detected with resnet convolutional neural network [16], [17] to add in dataset short circuit and drop detachment frequency. This molten volume was calculated applied equations shown in [12].

## CRediT Author Statement

**Rogfel Thompson Martinez:** Visualization, Conceptualization Methodology, Software; **Guillermo Alvarez Bestard:** Resources, Software, Validation, Supervision; **Sadek C. Absi Alfaro:** Investigation, Validation, Supervision.

## Declaration of Competing Interest

The authors declare that they have no known competing financial interests or personal relationships, which have or could be perceived to have influenced the work reported in this article.
